# Association between dynamic fluctuations in triiodothyronine levels and prognosis among critically ill patients within comprehensive intensive care units

**DOI:** 10.3389/fendo.2023.1282547

**Published:** 2023-11-29

**Authors:** Yu Xu, Kang Xu, Jianying Guo, Mingxing Fang, Zhiyong Wang

**Affiliations:** Department of Critical Care Medicine, Hebei Medical University Third Hospital, Shijiazhuang, China

**Keywords:** severe disease, free thyroid hormone (FT3), dynamic, SOFA score, prognosis

## Abstract

**Objective:**

Decrease in free thyroid hormone T3 (FT3) can be used as an independent prognostic indicator for the risk of death in ICUs. However, FT3 as a predictive marker is hindered by its accuracy. The study introduces the concept of dynamic FT3 data as a means to bolster the value of FT3 as a prognostic tool. Therefore, the aim of this study is to investigate the prognostic value of dynamic FT3 evolution in a comprehensive ICU setting, analyze the consistency between dynamic FT3 changes and variations in disease severity, and explore the feasibility of FT3 as an objective indicator for real-time clinical treatment feedback.

**Methods:**

Employing a single-center prospective observational study, FT3 measurements were taken on multiple days following enrollment, corresponding clinical data were collected. To investigated the pattern of dynamic changes of FT3,its prognostic significance in forecasting the risk of 28-day mortality, the alignment between dynamic FT3 changes and variations in the Sequential Organ Failure Assessment (SOFA) score.

**Results:**

The survival group exhibited higher last FT3 levels compared to the lowest point (p<0.05), while the death group did not show statistically significant differences (p>0.05). The study also identifies the optimal correlation between FT3 and SOFA score at day 5 (optimal correlation coefficient -0.546).The ROC curve for FT3 at day 5 yielded an optimal AUC of 0.88, outperforming the SOFA score. The study categorizes FT3 curve patterns,Kaplan-Meier survival analysis of these patterns highlighted that the descending-type curve was significantly associated with increased risk of death (P<0.001). Additionally, the research explores the consistency between changes in FT3 and SOFA scores. While overall consistency rates were modest, subgroup analyses unveiled that greater disease severity led to higher consistency rates.

**Conclusions:**

This study introduces the concept of dynamic FT3 changes to augment its prognostic utility in comprehensive ICU settings. The research identifies day 5 as the optimal time point for predictive efficacy, the descending FT3 curve as indicative of poor prognosis. While overall consistency with SOFA scores is modest, the correlation strengthens with greater disease severity.

## 1 Introduction

ICU patients, often in critical situation characterized by a significant mortality rate, require prompt and accurate assessment of disease severity and prognosis. This necessitates the ICU’s ability to swiftly predict and evaluate outcomes. Presently, the widely used metrics for disease severity and prognosis in ICUs are the Acute Physiology And Chronic Health Evaluation II (APACHE II)score and Sequential Organ Failure Assessment (SOFA) score (1, 2). The diagnosis of sepsis 3.0 is based on infection criteria coupled with a SOFA score greater than 2 (3). However, the complex scoring criteria for APACHE II and SOFA scores entail collecting numerous clinical and laboratory indicators, rendering the scoring process intricate. Moreover, it can be influenced by factors like sedation and medical interventions, leading to evaluator subjectivity (4). Consequently, these scores are primarily utilized for medical quality control and scientific research, rather than for timely and practical clinical applications in assessing disease outcome.

In comparison to these diverse scoring systems, a singular, rapid, and objective index holds advantages for disease severity assessment and prognosis. Abundant research underscores the association of free thyroid hormone T3 (FT3) with the prognosis of various conditions, including sepsis, heart failure, myocardial infarction, COVID-19, cardiomyopathy, liver failure associated with hepatitis B virus, and pulmonary arterial hypertension (5–11). FT3 has emerged as an independent prognostic indicator for ICU patients. Studies have shown that ESS also has a high mortality risk in critically ill children (12, 13), but the area under the curve of FT3 is 0.644 (95% confidence interval 0.555-0.789). The area under the curve (AUC) of FT4 is 0.655 (0.56-0.78, p = 0.02), with a sensitivity of 76% and a specificity of 61.5% for detecting high mortality risk. The predictive performance is low. In 2020, our research team conducted a prospective observational study involving 305 comprehensive ICU patients (14), demonstrating the potential of FT3 as an independent prognostic marker. However, the ROC curve’s AUC value for FT3 in relation to 28-day mortality was 0.671, corresponding to 81.5% specificity and only 51.4% sensitivity. Most studies, including our own, have utilized FT3 data within 24 hours of ICU admission for prognostic evaluation, without monitoring dynamic FT3 changes. Considering the possibility of enhanced prognostic value, it’s worth exploring the potential impact of dynamic FT3 data. Additionally, the connection between dynamic FT3 changes and alterations in disease severity remains crucial. If dynamic FT3 changes align with shifts in disease severity, FT3 could serve as an objective marker, providing real-time feedback on treatment efficacy—an invaluable asset in critical patient care.

Hence, this research project aims to conduct a prospective observational study, investigating dynamic FT3 changes and their correlation with comprehensive ICU patient prognosis. The objective is to ascertain the significance of dynamic FT3 in prognostic assessment, delve into the relationship between dynamic FT3 changes and shifts in disease severity, and determine whether dynamic FT3 variations offer real-time feedback on ICU treatment effectiveness. These insights would serve as a guide for informed clinical decision-making.

## 2 Materials and methods

### 2.1 Inclusion and exclusion criteria

The study’s inclusion criteria encompassed: (1) individuals aged over 18 years; (2) patients admitted to the ICU for various reasons; (3) absence of prior abnormal thyroid function or thyroid tumor history. Conversely, exclusion criteria involved: (1) patients with ICU stays shorter than 5 days or fewer than 3 FT3 measurements; (2) organ donors; (3) pregnant or lactating women.

### 2.2 Data collection and examination procedures

The research encompassed collecting general information, clinical examination outcomes, diagnostic and therapeutic details from enrolled patients. This encompassed aspects like gender, age, vital sign data, and free thyroid function (FT3, FT4, TSH), as well as biochemical, blood routine, and blood gas analysis findings on days 1, 3, 5, 7, 9, and 14. Moreover, Acute Physiology and Chronic Health Evaluation II score (APACHE II), Glasgow Coma Score (GCS), and Sequential Organ Failure Score (SOFA) were used to evaluate patients’ conditions.

Nurse-administered blood gas analysis tests were conducted in the ICU, involving arterial blood samples analyzed with a multifunction blood gas analyzer (Rayto, USA). The Department of Nuclear Medicine (Beckman DXI800, USA) at our hospital employed radioimmunoassay for thyroid function assessment. Normal reference values were: FT3 (3.53 ~ 7.37 pmol/L), FT4 (7.98 ~ 16.02 pmol/L), and TSH (0.56 ~ 5.91 mIU/L). Remaining laboratory indices were measured by the Department of Laboratory Medicine at our hospital.

Patients were categorized based on FT3 level changes into the FT3 normal group, FT3 U-type group (FT3 increased by over 15% after reaching the lowest point), and FT3 decreasing group (FT3 fluctuated or did not increase by 15% after reaching the lowest point) (15). Admission date, lowest point, and lastpoint values following enrollment were extracted. Prognosis (survival or death) within 28 days post-enrollment was obtained from our hospital’s HIS system or via telephone follow-up.

It’s noteworthy that low triiodothyronine (T3) levels, decreased or normal thyroxine (T4) and thyroid-stimulating hormone (TSH), and increased reverse triiodothyronine (rT3) levels are associated with acute illness-related changes in serum thyroid function, termed “normal thyroid disease syndrome ESS” (14).

### 2.3 Statistical analysis

Statistical analysis was conducted using SPSS version 27.0. Categorical variables were presented as numbers (percentage, %) and compared using the χ^2^ test. Normally distributed continuous variables were expressed as mean ± standard deviation ( $\bar{\text{X}}$ ± SD). The t-test was employed to compare means between two groups, while analysis of variance (ANOVA) was utilized for comparing means among three groups. In instances of skewed distributions, continuous variables were represented by the median and interquartile range (IQR), with the Kruskal-Wallis rank sum test applied for comparison. Correlations between variables were explored using Spearman’s coefficient. For survival analysis, Kaplan-Meier curves were generated and cumulative survival analysis was conducted through the log-rank test. The predictive risk of FT3 for mortality was evaluated using the receiver operating characteristic (ROC) curve. Multifactorial logistic regression analysis was employed to identify risk factors. Statistically significant differences were considered for p-values below 0.05.

## 3 Results

### 3.1 Enrollment characteristics

**Figure 1:**
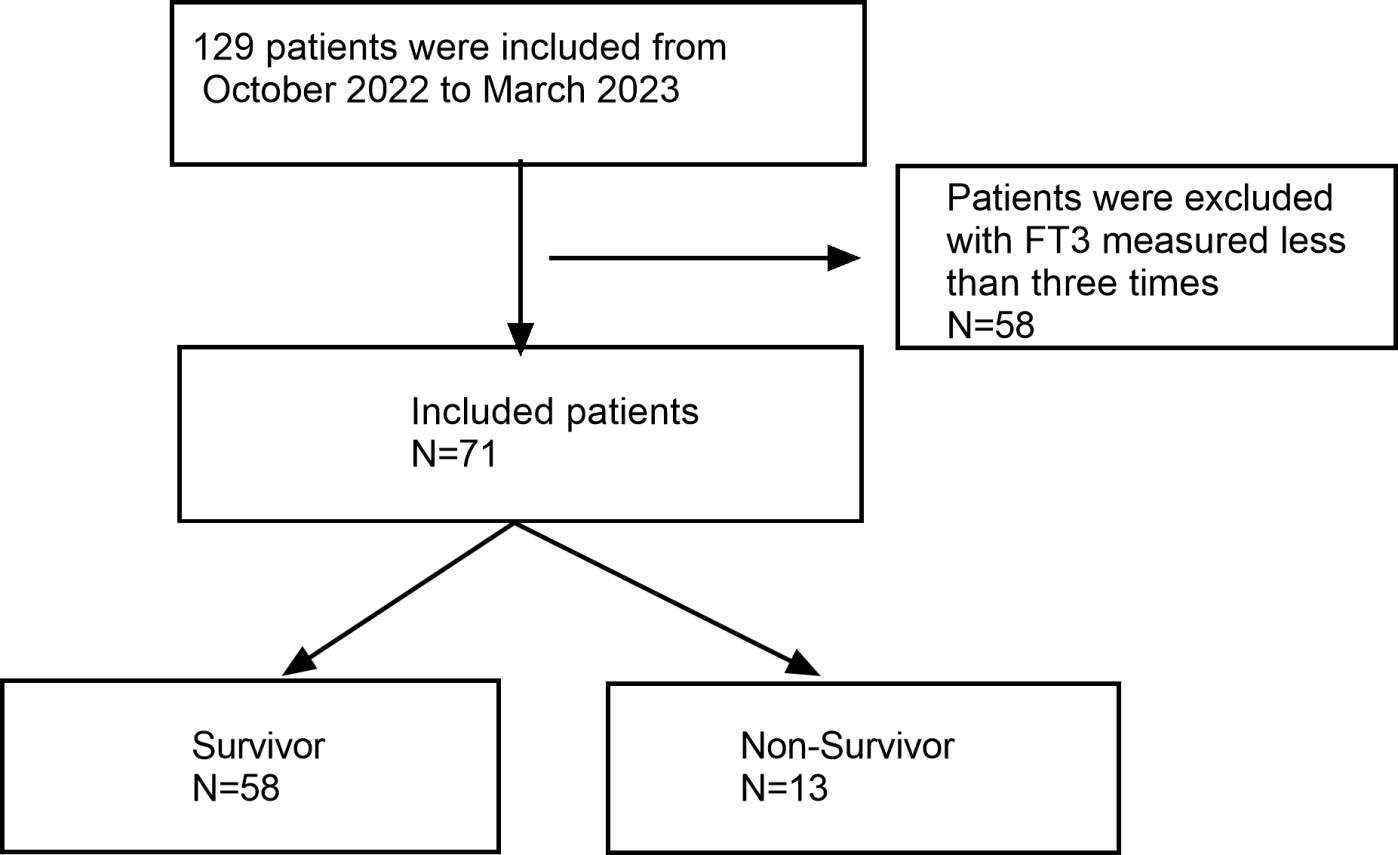
Flow chart of patients in the study cohort.

Among 129 initially screened patients, 71 eligible patients were included, comprising 13 deaths and 58 survivors. The flowchart is depicted in **Figure 1**. Of these, 54 (76.1%) consisted of males with a mean age of 60.95 ± 17.99 years, and 17 (23.9%) consisted of females with a mean age of 66.55 ± 19.01 years. **Table 1** showcases the characteristics of patients in the survival and death groups upon enrollment. Notably, there were notable differences between the two groups in Heart rate(HR), APACHE II, SOFA, GCS, FT3, FT4, Albumin (ALB), Lactic acid (Lac), Blood platelet (PLT), Creatinine (CREA), and Oxygenation index (PaO2/FIO2), with FT3 and FT4 levels being lower in the death group compared to survivors (P < 0.05). However, TSH showed no significant distinction between the two groups (P = 0.167).

**Table 1:** Patient demographic information at admission.

Variable	Survival group		Non-survival group	P-value
	58cases		13cases	
Clinical characteristics				
Age (years)	60.95 ± 17.99		66.55 ± 19.01	0.342
Gedner type				0.948
Female	14 (24.14%)		3 (23.08%)	
Male	44 (75.86%)		10 (76.92%)	
Vital signs when enrolled				
HR	82.3 ± 25.4		103.0 ± 31.5	0.013
T (°C)	37.18± 0.66		37.42 ± 1.01	0.277
RR	21.10 ± 11.33		19.62 ± 5.56	0.647
MAP (mmHg)	86.31 ± 21.27		81.23 ± 24.17	0.450
Laboratory test				
FT4 (pmol/L)	12.89 ± 4.53		9.52 ± 4.33	0.017
FT3 (pmol/L)	3.89 ± 1.15		3.07 ± 1.10	0.023
TSH (mIU/L)	2.04 ± 2.64		1.35 ± 1.36	0.167
ALB (g/L)	30.94 ± 6.06		26.18 ± 6.04	0.013
ALT (mmol/L)	32.00 (18.00-66.75)		49.00 (25.00-100.00)	0.334
TBIL (mmol/L)	17.45 (10.96-24.29)		23.07 (12.88-59.09)	0.133
CREA (umol/L)	66.05 (54.17-95.34)		146.58 (103.39-286.40)	<0.001
K (mmol/L)	3.99 (3.75-4.23)		3.70 (3.68-4.51)	0.645
Na (mmol/L)	134.04 ± 24.45		139.05 ± 5.32	0.467
WBC (×10^9^/L)	9.84 (7.72-12.43)		13.65 (6.51-17.35)	0.193
HGB (g/L)	100.62 ± 18.25		93.23 ± 20.01	0.199
PLT (×10^9^/L)	189.50 (107.00-257.00)		50.00 (34.00-150.00)	0.006
Lactic acid (mmol/L)	1.37 ± 0.84		2.58 ± 1.64	0.001
Oxygenationindex(PaO2/FIO2)	238.76 ± 87.48		165.29 ± 79.53	0.007
Prognostic score				
SOFA mean ± SD	7.43 ± 3.52		13.15 ± 3.56	<0.001
GCS mean ± SD	10.41 ± 3.41		7.69 ± 3.57	0.012
APACHEII mean ± SD	16.71 ± 7.31		26.62 ± 6.37	<0.001

T, temperature; HR, heart rate; MAP, mean arterial pressure; RR, respiratory rate; FT3, free triiodothyronine; FT4, free thyroxine; TSH, thyroid-stimulating hormone; ALT, alanine aminotransferase; ALB, albumin; WBC, white blood cell; HGB, hemoglobin; PLT, blood platelet; CREA, creatinine; TBil, total bilirubin; APACHE II score, acute physiology and chronic health evaluation II score; GCS, Glasgow coma scale; SOFA, sequential organ failure score.

### 3.2 Longitudinal changes in free thyroid function and prognostic scores

**Table 2** outlines the changes in FT3 levels and prognostic scores. At 1d, 3d, 5d, and 7d, the death group displayed lower FT3 and FT4 levels along with reduced GCS scores compared to the survival group. Conversely, the death group exhibited higher APACHE II and SOFA scores than the survival group.

**Table 2:** Free thyroid function and prognostic score at D1、D3、D5、D7、D9、D14.

Variable	Total (n=71)	Survival grpoup (n=58)		Non-survival group (n=13)		P-value
	Patients with available data	Patients with available data	Median± standard	Patients with available data	Median± standard	
FT3 (pmol/L)						
D1	n=71	n=58	3.89 ± 1.15	n=13	3.07 ± 1.10	0.023
D3	n=71	n=58	3.86 ± 1.21	n=13	2.84 ± 0.84	0.005
D5	n=71	n=58	3.96 ± 0.95	n=13	2.88 ± 0.50	<0.001
D7	n=51	n=43	3.82 ± 0.98	n=8	2.83 ± 0.54	0.008
D9	n=32	n=29	3.65 ± 0.71	n=3	3.85 ± 0.55	0.641
D14	n=24	n=24	4.09 ± 1.19	n=0		
FT4 (pmol/L)						
D1	n=71	n=58	12.89 ± 4.53	n=13	9.52 ± 4.33	0.017
D3	n=71	n=58	12.98 ± 3.71	n=13	10.08 ± 4.28	0.016
D5	n=71	n=58	12.85 ± 3.19	n=13	9.59 ± 4.87	0.004
D7	n=51	n=43	12.62 ± 3.12	n=8	8.04 ± 2.22	<0.001
D9	n=32	n=29	12.41 ± 3.74	n=3	7.49 ± 3.81	0.038
D14	n=24	n=24	12.58 ± 3.10	n=0		
TSH (mIU/L)						
D1	n=71	n=58	2.04 ± 2.64	n=13	1.35 ± 1.36	0.167
D3	n=71	n=58	2.05 ± 1.73	n=13	1.21 ± 1.38	0.04
D5	n=71	n=58	2.23 ± 2.03	n=13	0.49 ± 0.45	<0.001
D7	n=51	n=43	2.64 ± 1.97	n=8	0.65 ± 0.839	0.007
D9	n=32	n=29	2.14 ± 1.35	n=3	1.15 ± 1.34	0.241
D14	n=24	n=24	2.42 ± 1.59	n=0		
APACHEII						
D1	n=71	n=58	16.71 ± 7.31	n=13	26.62 ± 6.37	<0.001
D3	n=71	n=58	15.98 ± 6.47	n=13	25.92 ± 5.79	<0.001
D5	n=71	n=58	16.19 ± 7.08	n=13	26.69 ± 7.15	<0.001
D7	n=51	n=43	16.37 ± 6.39	n=8	26.38 + 7.25	<0.001
D9	n=32	n=29	17.34 ± 7.54	n=3	29.33 ± 8.15	0.014
D14	n=24	n=24	17.63 ± 7.67	n=0		
SOFA						
D1	n=71	n=58	7.43 ± 3.52	n=13	13.15 ± 3.56	<0.001
D3	n=71	n=58	7.16 ± 3.59	n=13	13.54 ± 3.97	<0.001
D5	n=71	n=58	6.98 ± 3.80	n=13	13.77 ± 5.02	<0.001
D7	n=51	n=43	6.47 ± 3.54	n=8	15.00 ± 4.69	<0.001
D9	n=32	n=29	6.93 ± 3.97	n=3	17.33 ± 4.62	<0.001
D14	n=24	n=24	6.58 ± 3.41	n=0		
GCS						
D1	n=71	n=58	10.41 ± 3.41	n=13	7.69 ± 3.57	0.012
D3	n=71	n=58	10.59 ± 3.36	n=13	6.54 ± 3.28	<0.001
D5	n=71	n=58	10.81 ± 3.31	n=13	5.92 ± 3.84	<0.001
D7	n=51	n=43	8.05 ± 5.25	n=8	3.38 ± 4.41	0.004
D9	n=32	n=29	10.72 ± 3.15	n=3	5.33 ± 4.04	0.009
D14	n=24	n=24	10.38 ± 2.96	n=0		

FT3, free triiodothyronine; FT4, free thyroxine; TSH, thyroid-stimulating hormone; SOFA, sequential organ failure assessment; APACHE II, Acute Physiology and Chronic Health Evaluation II; GCS, Glasgow coma scale.

### 3.3 Dynamic FT3 changes in survival and death groups

**Figure 2:**
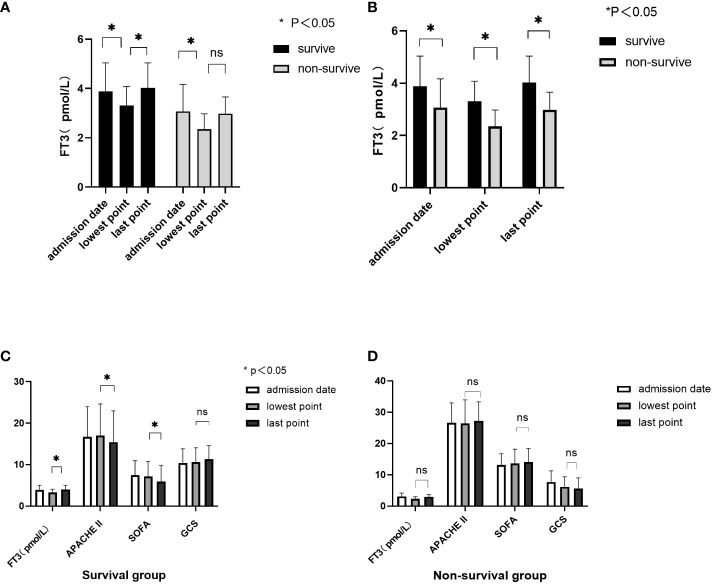
Correlation between FT3 dynamic change and prognostic score. **(A, B)** Characteristics of changes in FT3 at admission, the lowest point, and the last point; **(C)** FT3 levels and prognostic score changes in the survival group; **(D)** FT3 levels and prognostic score changes in the non-survival group. ns, no significant difference.

The lowest FT3 levels were observed around 3 (1-6) days after enrollment within the 1 to 14-day range. **Figure 2** presents the admission, lowest, and lastpoint FT3 values, alongside relevant prognostic score characteristics in the context of survival and death groups. The survival group demonstrated significantly higher admission, lowest, and lastpoint FT3 levels compared to the death group (P values: P = 0.028, P < 0.001, and P < 0.001, respectively). Notably, in the survival group, the lastpoint FT3 level surpassed the lowest level, accompanied by reduced APACHE II and SOFA scores (P < 0.01). Conversely, changes in FT3 and corresponding prognostic scores in the death group between lastpoint and lowest values were not statistically significant (P > 0.05).

### 3.4 Relationship between changing FT3 levels and prognostic scores

**Table 3** showcases the correlation analysis between FT3 levels on various days (D1, D3, D5, and lowest Point) and APACHE II and SOFA scores. Compared with D1 and D3, FT3 displayed the strongest correlation with APACHE II and SOFA scores on day 5, with respective correlations of -0.505 and -0.546.

**Table 3:** Correlation between FT3 level at D1、D3、D5、lowest and Relevant conventional prognostic score.

	R	P value
APACHEII		
D1	-0.309	0.009
D3	-0.411	<0.001
D5	-0.505	<0.001
lowest	-0.52	<0.001
SOFA		
D1	-0.34	0.004
D3	-0.387	<0.001
D5	-0.546	<0.001
lowest	-0.542	<0.001

SOFA, sequential organ failure assessment; APACHE II, Acute Physiology and Chronic Health Evaluation II.

### 3.5 ROC Curve analysis of dynamic FT3 and prognostic scores for 28-day mortality

**Figure 3:**
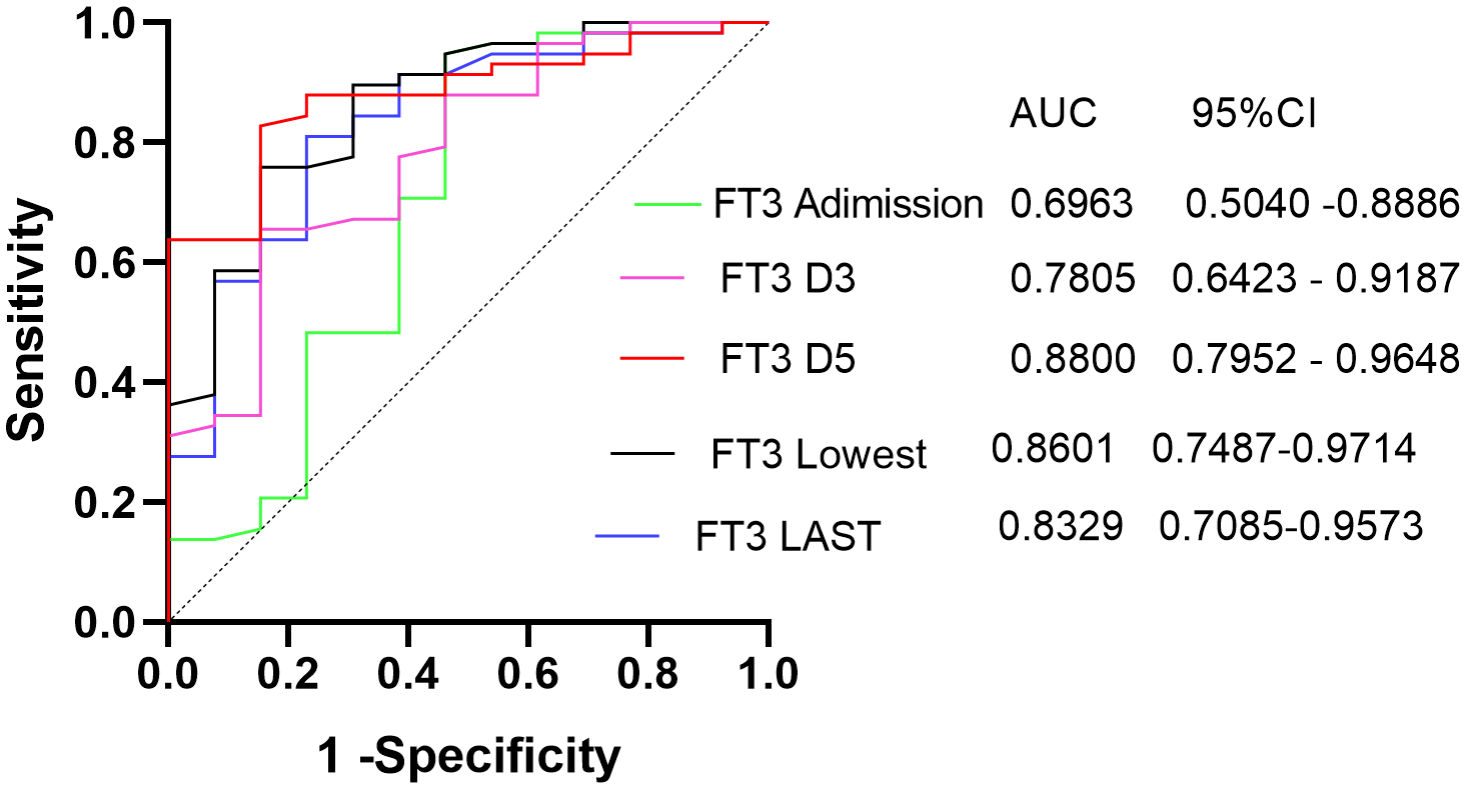
ROC curves of FT3 as an overall predictor of death.

**Figure 4:**
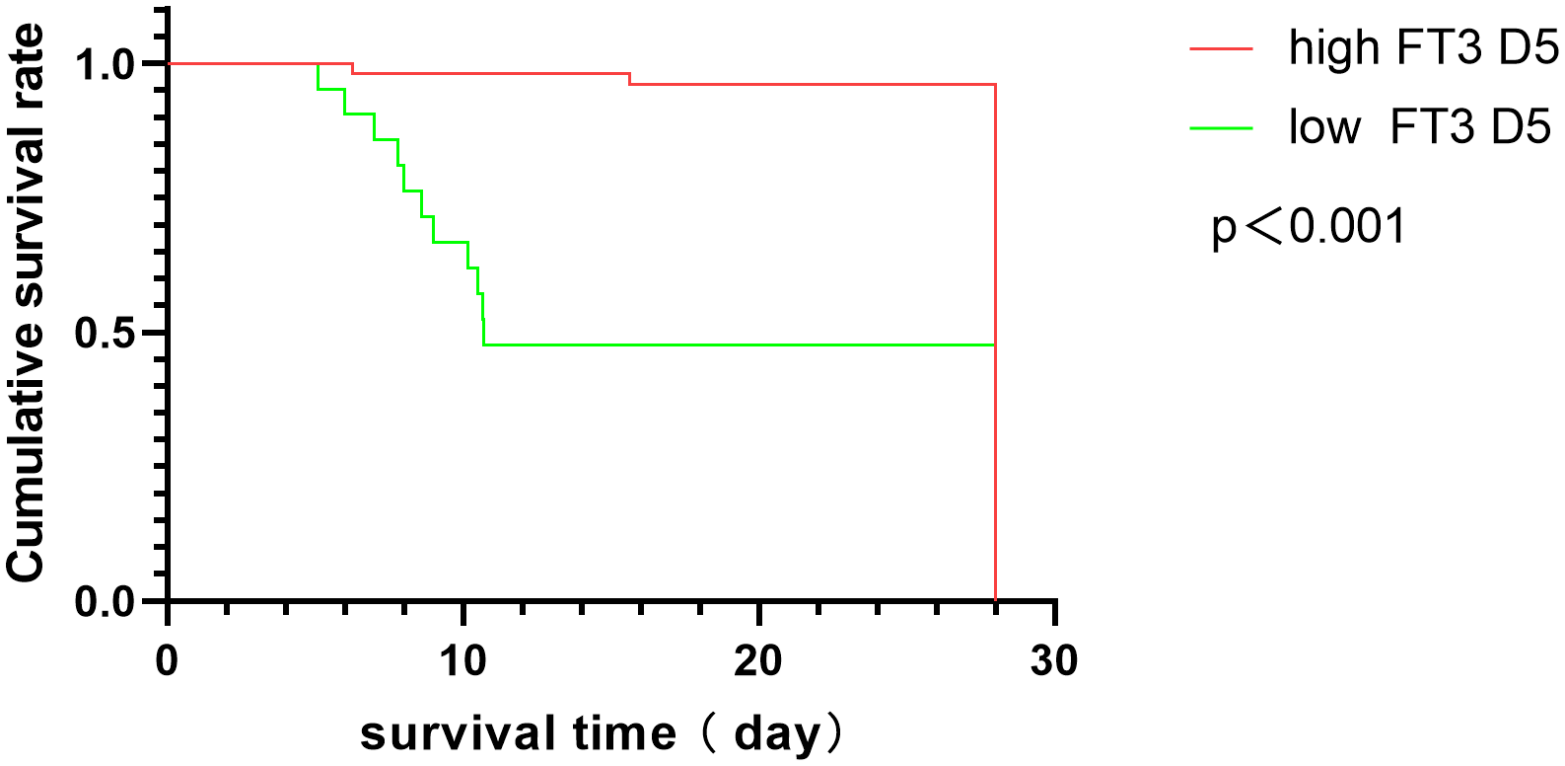
Kaplan-Meier survival curves in ICU patients with high and low FT3 at D5. A log-rank test was used to evaluate differences between groups.

**Figure 3** illustrates the ROC curve analysis, indicating that the area under the curve (AUC) for FT3 was 0.69 at enrollment and exceeded 0.7 on days D3, D5, lowest Point, and Last Point. The highest AUC was observed on day 5 for FT3, at 0.88. Additionally, APACHE II score and SOFA score AUCs were 0.85 and 0.86, respectively, on day 5. The optimal FT3 cutoff value was 3.305 pmol/L, yielding a sensitivity of 0.8276 and specificity of 0.8462. Kaplan-Meier survival curves, based on the 5th day FT3 cutoff, revealed higher 28-day mortality in the low FT3 level group (p < 0.001), with an OR value of 9.96 (**Figure 4**).

### 3.6 Factors associated with 28-day mortality rate

Univariate analysis demonstrated that 5th-day FT3, APACHE II score, SOFA score, Lac, and ALB were linked to 28-day prognosis (**Table 4**). Multivariate logistic regression analysis confirmed that 5th-day FT3 remained an independent factor influencing 28-day mortality after adjusting for variables with P < 0.05 in univariate analysis (OR = 0.049, 95% CI: 0.005-0.476, P = 0.009) (**Table 4**).

**Table 4:** Univariable and multivariable logistic regression analyses for 28d mortality.

		OR	Univariable 95%CI	P	OR	Multivariable 95%CI	P
D5	FT3	0.092	(0.024,0.35)	<0.001	0.049	(0.005,0.476)	0.009
	SOFA	1.372	(1.162,1.619	<0.001	1.233	(1.024,1.486)	0.027
	APACHEII	1.213	(1.092,1.348)	<0.001			
	ALB	0.904	(0.832,0.981)	0.016	0.773	(0.641,0.932)	0.007
	Lactic acid	4.612	(1.689,12.592)	0.003			

Variables included in the multivariable logistic analysis wereFT3, ALB, APACHEII, SOFA and Lac. FT3, free triiodothyronine; APACHE II score, acute physiology and chronic health evaluation II score; SOFA, sequential organ failure score; ALB: albumin.

### 3.7 Types of dynamic FT3 changes and patient prognosis

**Figure 5:**
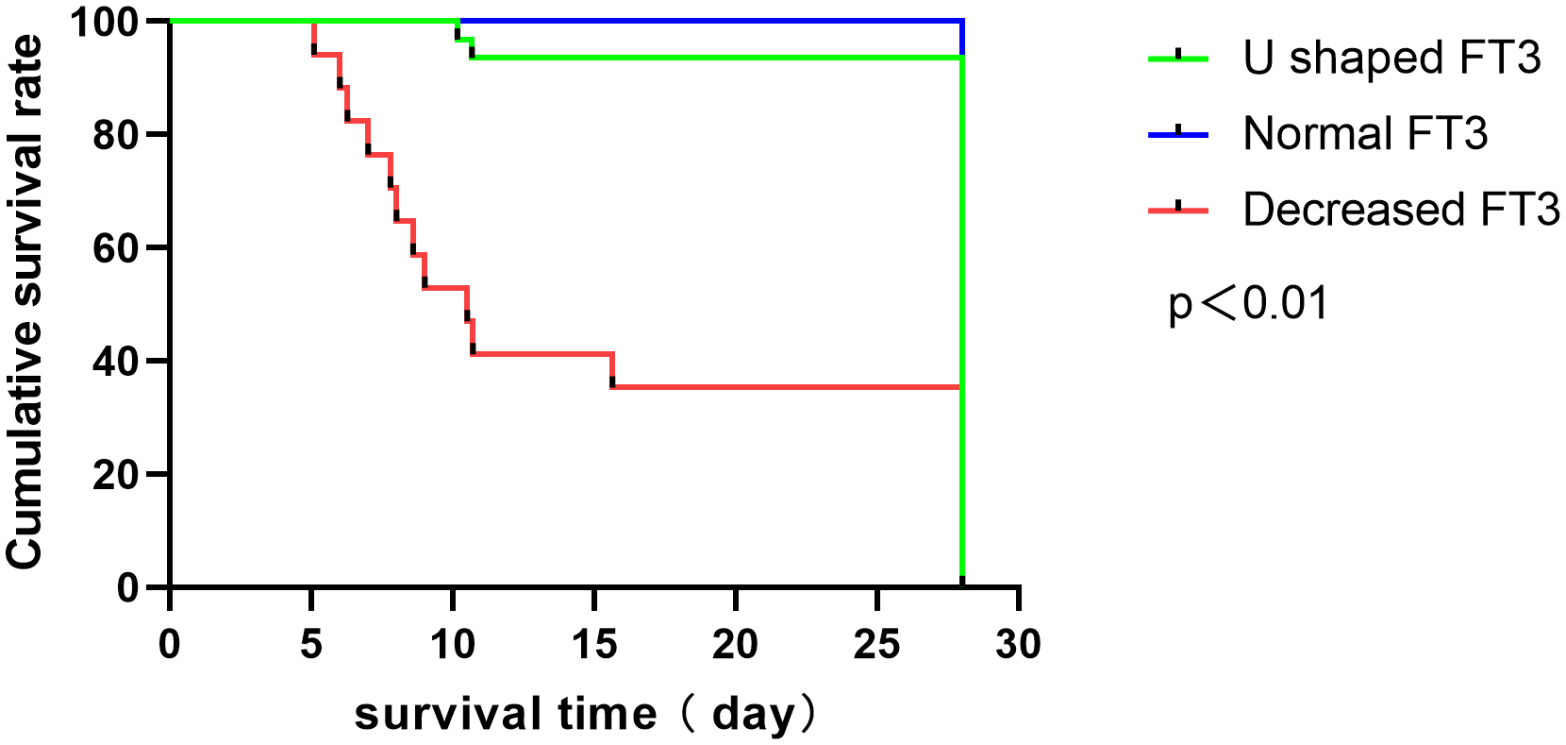
Survival analysis function of patients with different types of FT3 level changes.

Patients were categorized based on FT3 changes into normal-type (23 cases, 32.39%), descending-typel (16 cases, 22.54%), and U-shaped change groups (32 cases, 40.57%). Kaplan-Meier survival analysis revealed significantly increased risk of death in the decreasing-type group compared to normal and U-type groups (P < 0.001), with an OR of 26.58 (**Figure 5**).

### 3.8 Consistency of real-time FT3 changes with SOFA score changes

Consistency between FT3 and SOFA score changes was observed at d3-1 and d5-3. This consistency indicated that an increase in SOFA score corresponded with FT3 decrease and vice versa. The findings are detailed in **Table 5**. Among the patients, 31 cases (43.6%) exhibited concordant changes at d3-1, while 32 cases (44.4%) displayed concordant changes at d5-3.

**Table 5:** Consistent change with FT3 and SOFA.

	Total (case)	Covariance population (case)	
		△d3-1	△d5-3
All	71	31	32
ESS	35	17	16
Non-survival	13	7	4
SOFA>10	20	13	8

ESS, euthyroid sick syndrome; ALL, all patients.

Subgroup analysis of the patient cohort revealed the following: in ESS patients, 17 cases (48.6%) exhibited concordant changes in d3-1 and 16 cases (45.7%) in d5-3; among dead patients, 7 cases (53.8%) had concordant changes in d3-1 and 4 cases (30.8%) in d5-3; additionally, when the SOFA score was over 10, there were 13 cases (65.0%) of concordance in d3-1 and 8 cases (40%) in d5-3.

**Figure 6:**
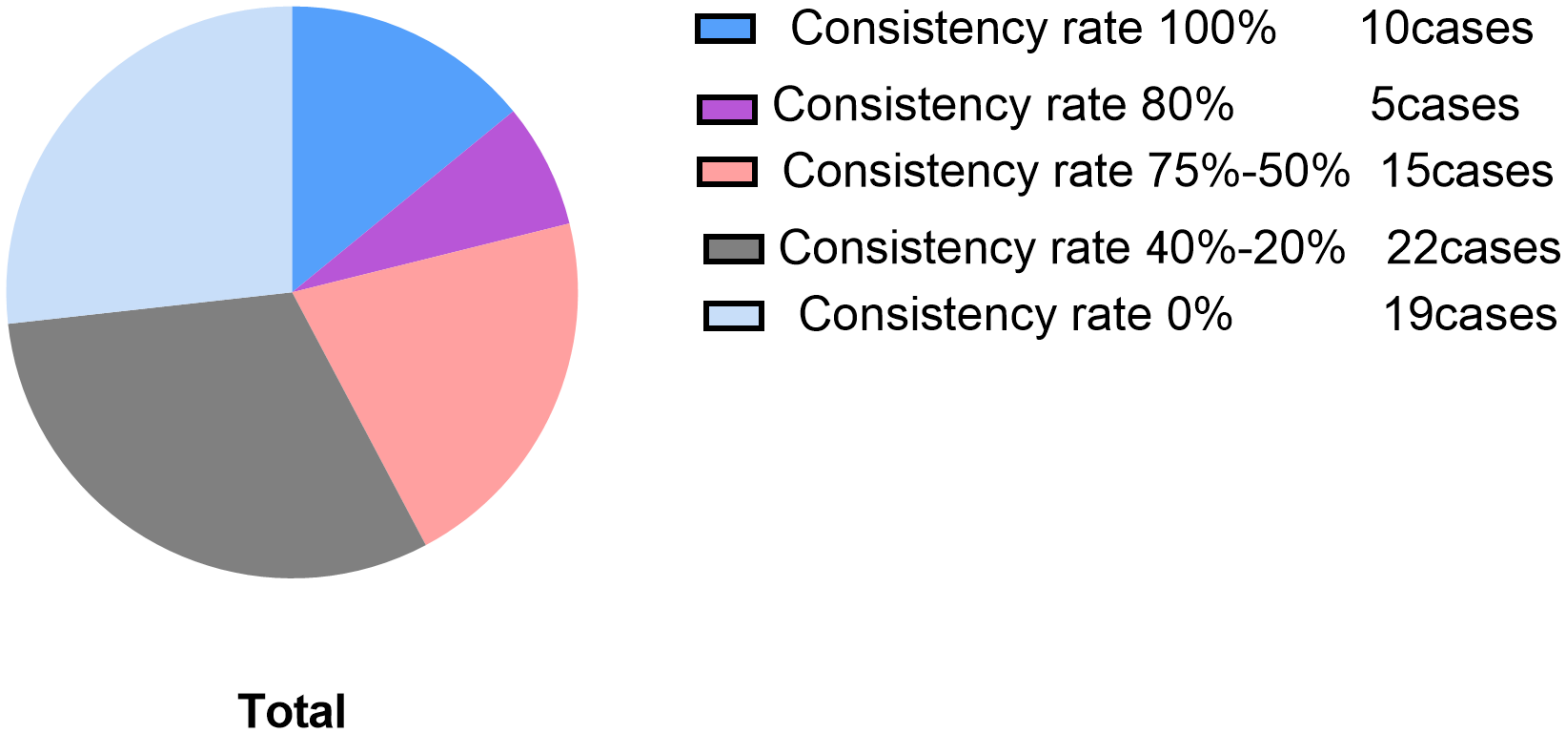
Consistency rate pie chart of total population.

**Figure 7:**
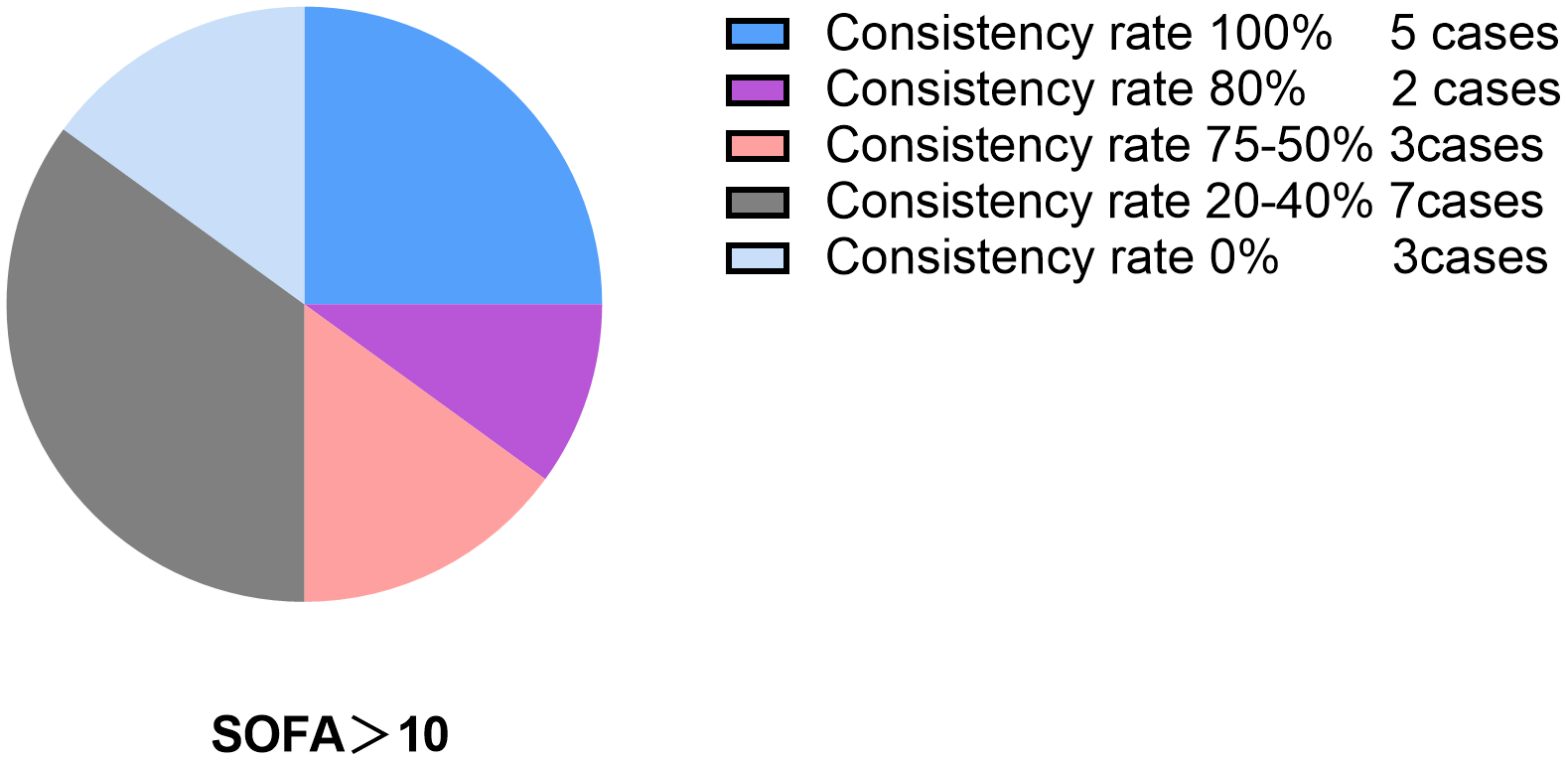
Consistency rate pie chart when SOFA>ten point.

The coherence between alterations in FT3 levels and SOFA scores was observed individually within all 71 enrolled patients. Notably, in 15 patients, the coherence exceeded 80%, with a complete coherence rate of 100% in 10 patients, as depicted in **Figure 6**. Among the subset of patients with SOFA scores >10 (a total of 20 patients), coherence surpassed 80% in 7 patients, with a complete coherence rate of 100% in 5 patients, as illustrated in **Figure 7**.

## 4 Discussion

This study delved into the prognostic significance of dynamic FT3 changes among ICU patients. The findings revealed that 49.3% of ICU patients exhibited Euthyroid Sick Syndrome (ESS), aligning with previous research encompassing 305 comprehensive ICU patients, which established ESS incidence within the range of 32.8% to 44.6% (16). This consistency provides a foundation for utilizing FT3 levels as prognostic indicators for ICU patient outcomes. Furthermore, the present study aligned with comprehensive related research, indicating that FT3 levels at ICU admission were significantly lower in deceased patients than in survivors (17, 18). The pathogenesis of Euthyroid Sick Syndrome (ESS) in critically ill patients is a complex process characterized by the suppression of hypothalamic thyrotropin-releasing hormone, persistent reduction in thyroid-stimulating hormone secretion despite low plasma thyroid hormone levels, altered expression and activity of thyroid hormone metabolism enzymes (D1, D2, and D3), increase of D3、decreased of D2, reduced thyroid hormone transporter levels, specific changes in thyroid hormone receptors, and the influence of inflammatory cytokines. These disruptions collectively lead to decreased plasma T3 levels during acute illness, particularly in the presence of an inflammatory response (19, 20).

Notably, this study tracked longitudinal FT3 changes alongside prognostic scores, revealing that in the death group, FT3 and FT4 levels as well as GCS scores were consistently lower compared to those in the survival group on days 1, 3, 5, and 7. Meanwhile, APACHE II and SOFA scores were consistently higher in the death group. These trends underscore the close association between persistently low FT3 levels and prognosis. While the 9th-day FT3 in the death group was higher than the survival group, this difference didn’t reach statistical significance. This could be attributed to the small sample size and individual variations, necessitating larger-scale studies to explore these trends further. Additionally, In order to further analyze the relationship between dynamic changes in FT3 and the risk of death, investigating FT3 levels at admission, lowest point, and lastpoint values unveiled that the survival group had significantly higher admission, lowest, and lastpoint FT3 levels compared to the death group, reinforcing the link between persistently low FT3 and prognosis. In the survival group, increasing FT3 levels were associated with declining APACHE II and SOFA scores (P < 0.01). This alignment between FT3 changes and disease severity-driven prognostic score shifts suggests that dynamic FT3 changes might influence the 28-day prognosis of critically ill patients.

Classification based on FT3 change trends resulted in the FT3 normal-type, FT3 decreasing-type, and FT3 U-shaped change group. Kaplan-Meier survival analysis underscored a significantly higher risk of death in the decreasing group, reaffirming that lower FT3 levels correlate with elevated mortality rates. This finding aligns with Zhang J. et al.’s study on dynamic FT3 changes in patients with slow plus acute liver failure (15). However, differing viewpoints indicate that reduced thyroid hormone levels in critically ill patients are protective due to the lower metabolic rate and energy loss (21), contrary to this study’s findings. Further research is necessary to clarify these mechanisms.

Correlation analysis revealed that FT3 exhibited a robust correlation with prognostic scores (P < 0.05), and this correlation strengthened over time, with the strongest correlation observed between FT3 and SOFA scores on the 5th day. While previous studies found There was no significant correlation observed between initial FT3 levels at enrollment and most prognostic scores (15), these variations could arise from differences in disease severity, type, and applied prognostic scores. Unlike prior research that correlated FT3 within 24 hours of enrollment, this study explored dynamic FT3 correlations with prognostic scores. ROC curve analysis indicated that the predictive power of FT3 levels at enrollment was weak, with an AUC of 0.69. However, the AUC for FT3 at the 5th-day showing the highest AUC at 0.88, resulting in a sensitivity of 0.8276 and a specificity of 0.8462. surpassing APACHE II and SOFA scores on the same day. This emphasizes the improved accuracy of dynamic FT3 changes in prognosis determination, especially the sensitivity. Multivariate logistic regression further solidified 5th-day FT3 as an independent factor influencing 28-day mortality.

Concordance between FT3 and SOFA score changes on d3-1 and d5-3 was explored, demonstrating generally low alignment. Given SOFA score lag and the influence of medical interventions, particularly sedation and analgesia in ICU patients, neurological score accuracy might be compromised. Consequently, investigating alternative prognostic indicators in concordance with FT3 changes is warranted. Subgroup analyses suggested an increased concordance rate on d3-1 in ESS patients, dead patients, and those with SOFA scores >10. The consistency of FT3 and SOFA score changes individually was high in 15 patients, of which 46.7% had SOFA scores >10. This suggests that the more severe the disease, the timelier FT3 changes reflect disease fluctuations, a hypothesis warranting further exploration with larger sample sizes.

The study introduces the concept of dynamic FT3 changes to enhance its prognostic value in the context of comprehensive ICU settings. It identifies day 5 as the optimal time point for predictive efficacy and the descending FT3 curve as an indicator of poor prognosis, Significantly improved prediction of mortality risk. The following passage: Although the overall consistency with SOFA scores is low, it cannot negate the value of dynamic changes in FT3 in real-time response to disease severity. Soo A et al. (22) conducted a retrospective observational cohort study of critically ill patients, including 20,000 individuals, and performed ROC curve analysis on changes in SOFA scores at different time points. The results suggest a weak correlation between delta SOFA and mortality (AUC 0.56-0.62). It may be necessary to identify improved prognostic indicators for consistency analysis with FT3. Future research can focus more on enhancing the utilization of real-time feedback indicators for dynamic FT3 data. Once it can reflect prognosis, it will establish the foundation for clinical real-time feedback, paving the way for the development of more accurate ICU patient care prognostic tools. Additionally, FT3 and FT3 changes could be utilized as a variable to create a predictive model to forecast mortality. This study is a preliminary exploration and did not detect d2 and d4 FT3, nor did it conduct stratified analysis of disease types. Future multicenter studies should encompass diverse diseases and ensure sample balance.

## 5 Conclusion

Dynamic FT3 changes enhance the prognostic value of FT3 within comprehensive ICU contexts. The most effective predictive capability emerged at day 5, with persistently decreasing FT3 curves indicative of poor prognosis. While overall SOFA scores exhibited low alignment with FT3 changes, the concordance rate increased with disease severity. This study underscores the potential of dynamic FT3 as a prognostic indicator for critically ill patients’ outcomes.

## Ethical statement

The study was approved by the ethics committee of The Third Hospital of Hebei Medical University (No. 2018-019-1), and written informed consent was obtained.

## Data availability statement

The original contributions presented in the study are included in the article/Supplementary Material. Further inquiries can be directed to the corresponding author.

## Author contributions

YX: Writing – original draft, Writing – review and editing. KX: Writing – original draft. JG: Writing – review and editing. MF: Writing – review and editing. ZW: Writing – review and editing.
